# MIRIAM Resources: tools to generate and resolve robust cross-references in Systems Biology

**DOI:** 10.1186/1752-0509-1-58

**Published:** 2007-12-13

**Authors:** Camille Laibe, Nicolas Le Novère

**Affiliations:** 1 European Bioinformatics Institute, Wellcome Trust Genome Campus, Hinxton, Cambridge, UK

## Abstract

**Background:**

The *Minimal Information Requested In the Annotation of biochemical Models *(MIRIAM) is a set of guidelines for the annotation and curation processes of computational models, in order to facilitate their exchange and reuse. An important part of the standard consists in the controlled annotation of model components, based on Uniform Resource Identifiers. In order to enable interoperability of this annotation, the community has to agree on a set of standard URIs, corresponding to recognised data types. *MIRIAM Resources *are being developed to support the use of those URIs.

**Results:**

*MIRIAM Resources *are a set of on-line services created to catalogue data types, their URIs and the corresponding physical URLs (or resources), whether data types are controlled vocabularies or primary data resources. *MIRIAM Resources *are composed of several components: *MIRIAM Database *stores the information, *MIRIAM Web Services *allows to programmatically access the database, *MIRIAM Library *provides an access to the Web Services and *MIRIAM Web Application *is a way to access the data (human browsing) and also to edit or add entries.

**Conclusions:**

The project *MIRIAM Resources *allows an easy access to MIRIAM URIs and the associated information and is therefore crucial to foster a general use of MIRIAM annotations in computational models of biological processes.

## Background

Computational Systems Biology relies on developing large quantitative models of biological processes. Because of their size and complexity, those models need to be exchanged and reused, rather than rewritten. Standard formats have been created by the community to encode Systems Biology models, such as SBML [1], CellML [2] or BioPAX [3]. However, the fact that a model is syntactically correct does not ensure its semantic accuracy. Moreover, because of thematic or personal preferences, the terminology used to name model components varies widely. The community had therefore to define a set of guidelines to improve the quality of models aimed to be exchanged. The *Minimal Information Requested In the Annotation of biochemical Models *(MIRIAM) [4] fulfils this need by providing a standard for the annotation and curation of biochemical models.

MIRIAM is a project of the international initiative BioModels.net [5], which aims are multiple: define agreed-upon standards for model curation, define agreed-upon vocabularies for annotating models with connections to biological data resources and provide a free access to published, peer-reviewed, annotated, computational models. Others projects of this initiative includes BioModels Database [6], a free, centralised database of curated, published, quantitative kinetic models of biochemical and cellular systems; and the Systems Biology Ontology (SBO) [7]. All these projects together support the exchange and reuse of quantitative models. MIRIAM originates from the specific requirement to facilitate the exchange of kinetic models between databases, standards and software, as witnessed by the original authors, involved in BioModels Database, CellML, COPASI, DOCQS, JWS Online, MathSBML, RegulonDB, SBML, SBMLmerge, SBW and SigPath. The support of MIRIAM in the community has been growing steadily since its release, as witnessed by the growing number of citations, the recognition in community surveys [8] and the incorporation of MIRIAM annotations in widely used standard formats such as SBML [9]. Because quantitative modelling is only one facet of modern integrative biology, MIRIAM has now joined the Minimum Information for Biological and Biomedical Investigations (MIBBI), a broader effort to enhance cooperation between guidelines in life science [10].

An important part of MIRIAM requirements consists in the controlled annotation of model components, based on Uniform Resource Identifiers (URI) [11]. To summarise, all the components of a model need to be unambiguously identified in a *perennial *and *standard *way. This annotation should be consistent across all the data types used to annotate a model. *MIRIAM URIs *have been developed for this purpose. In this article we present the URI scheme used by MIRIAM annotations and the resources we have developed to support their usage by modellers and model users. Although these resources have been developed with the annotation of quantitative models in mind, they can be used as a generic resolving system for resources in biology.

## Construction and content

### MIRIAM URIs

An identifier is a single unambiguous string or label or name, that references or identifies an entity or object (that can be a publication, a database, a protein, a gene, etc.). The scientific community needs unique and perennial identifiers [12], to reliably describe, define or exchange objects, and therefore construct an integrated and fundamentally interoperable "bioinformatics world" [13].

An object identifier must be:

**Unique**: an identifier must never be assigned to two different objects;

**Perennial**: the identifier is constant and its lifetime is permanent;

**Standards compliant**: must conform to existing standards, such as URI;

**Resolvable**: identifiers must be able to be transformed into locations of on-line resources storing the object or information about the object;

**Free of use**: everybody should be able to use and create identifiers, freely and at no cost.

In addition an ideal identifier should be semantic-free, in the sense that it should not contain the information it is pointing to. A possible exception often mentioned are the InChIs [14], although this is debated. In particular they are not unique. Several objects can have the same InChI, for example of cis and trans-platin [15]. The precise form of InChI beyond the basic connectivity and stereochemistry layers depends on some parameters and different InChIs can be generated for the same compound. Finally, InChIs cannot be generated for some classes of compounds, for instance polymers.

Because of the perenniality requirement, one cannot use physical addresses, such as URLs [16] corresponding to physical documents, to reference pieces of knowledge. The use of numerical identifiers by themselves cannot be sufficient. "9606" represents *Homo sapiens *in the taxonomy databases, but a German article on social services for PubMed. Those identifiers of dataset acquire a meaning only within the context of a data type (generally, but not always, a given data resource). Some catalogues of data types in life science have been developed, recording the usual acronyms, such as the Gene Ontology database abbreviation [17]. However, the non-uniqueness of these acronyms makes them hardly usable. For instance, CGD is the acronym of the *Candida Genome Database*, but also the *Cattle Genome Database*. One approach to overcome this problem is to use unambiguous URI [11] instead. 

This approach has been successfully used for instance by the publishing industry with the Digital Object Identifier (DOI) [18,19] or by the astronomical community with the International Virtual Observatory Alliance (IVOA) Identifiers [20]. DOI have not been used widely in the scientific community because of the mandatory registration and their cost. Other generic systems of URI construction have been proposed such as the BioPAX URIs [21] or the PURL-based Object Identifier [22] (based on Persistent Uniform Resource Locator (PURL) [23] and Open Archive Initiative Identifiers [24]), but their structure does not allow to avoid the problems enumerated above. The closest effort to what is needed to annotate quantitative models are the Life Sciences Identifiers (LSID) [25,26]. And as a matter of fact, LSIDs are valid *MIRIAM URIs*. 

*MIRIAM URIs *are identifiers based on URI to uniquely refer to data entities. For more flexibility, they can follow two syntaxes: Uniform Resource Locator (URL) [16], like a common physical address on the Web, or Uniform Resource Name (URN) [27], like LSID. *MIRIAM URIs *are identifiers, as described previously, so they are unique, persistent, resolvable and freely usable. Moreover, they are case-sensitive, since URIs are. It is important to notice that, even when they comply with the URL scheme, they do not describe a physical resource, and several physical documents can present the information identified by one *MIRIAM URI*. Nevertheless, these physical locations can be retrieved by a resolution service described below. This feature is not unique, for example, DOI, PURL and LSID can be resolved through dedicated services. 

*MIRIAM URIs *are composed of two parts. First comes the URI of the data type, which is a unique, controlled description of the type of the data. For example, if the entity to annotate is a protein sequence, the data type could be UniProt. If the entity is an enzymatic activity, the data type could be the Enzyme Nomenclature of the International Union of Biochemistry and Molecular Biology, etc. The second part of the URI is the element identifier, which identify a specific piece of knowledge within the context of the data type.

As a result, a *MIRIAM URI *looks like:  <URI of the data type> # <identifier of the element> , summarised by <Authority> # <ID>. For example, in order to identify the publication describing MIRIAM, we can use: http://www.pubmed.gov/#16381840. Note that the "hash" is only necessary in the URL scheme but not in the URN one.

In order to enable interoperability of this annotation, the community has to agree on a set of recognised data types. *MIRIAM Resources *are an online service created to catalogue the data types, their URIs and the corresponding physical URLs or resources, whether these are controlled vocabularies or databases. Anybody can propose new data types that are included if they fulfil the necessary requirements of stability, openness and provide suitable identifiers and programmatic access.

**Figure 1:**
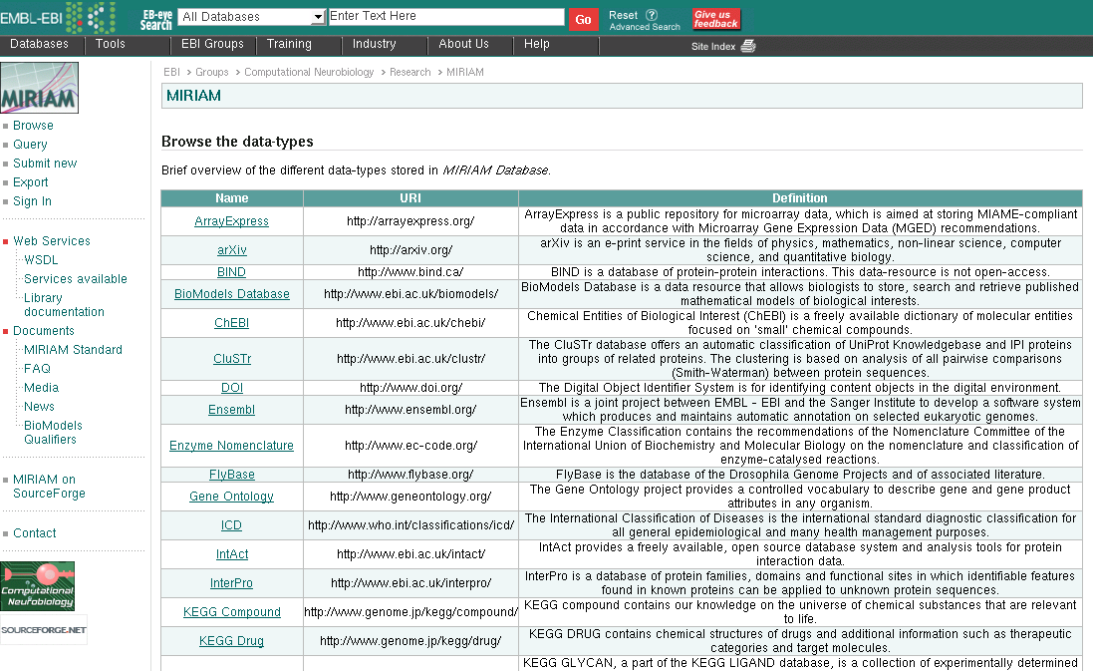
**MIRIAM Database browser**. Users can browse the content of *MIRIAM Database *and view the details of current entries, as well as propose new entries.

It is important to understand that MIRIAM data types do not represent kinds of biological information. They represent a standardised identification scheme for a type of biological information associated with a set of resources using the same set of identifiers. In some cases, different players in a domain agreed to unify the access to the data, such as PIR, SwissProt and TrEMBL with UniProt for protein sequences. In such a case, the data type corresponds mostly to the type of biological information. In other cases, several MIRIAM data types represent the same type of biological information presented independently by different resources. This is the case for chemical compounds for instance, for which MIRIAM uses ChEBI, KEGG Compound, PubChem Substance and Compound. See Figure 1 for a subset of the data types listed in *MIRIAM Database*.

*MIRIAM Resources *is therefore not designed to handle multiple aliases used to refer to a same biological information stored in different data resources using different identifiers. Other resources and tools already exist for that kind of purpose, such as *AliasServer *[28], *Sequence Globally Unique Identifiers *(SEGUID) [29] or the *International Protein Index *(IPI) [30] for protein sequences.

Moreover, MIRIAM data types do not *belong *to anybody, and in particular to the corresponding data providers.

**Figure 2:**
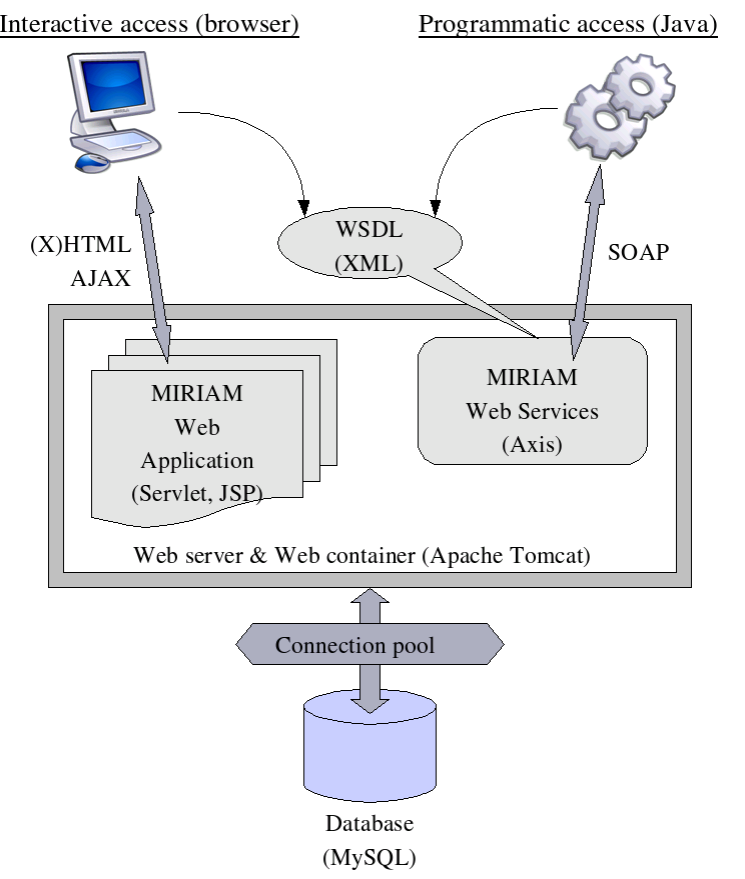
**Structure of MIRIAM Resources**. Diagram representing the different components of *MIRIAM Resources *and their relationships.

*MIRIAM Resources *is an open project, whether regarding its source code, the data stored and its access. It is divided into four components (Figure 2):

• *MIRIAM Database*: core element of the resource, storing all the information about the data types and their associated information;

• *MIRIAM Web Services*: SOAP-based application programming interface (API) for querying *MIRIAM Database*;

• *MIRIAM Library*: library to use *MIRIAM Web Services*;

• *MIRIAM Web Application*: interactive Web interface for browsing and querying *MIRIAM Database*, and also submit or edit data types.

All these components have been developed using the UTF-8 character encoding in order to allow the storage and display of international data. The usage of existing standards, where appropriate, has been preferred to enhance interoperability. Moreover, the project has been designed in order to allow its evolution and improvement, by including new data types to the database or by addition of new methods to the Web Services.

### MIRIAM Database

The core element of the resource is a relational database, using a MySQL database management system. The central elements are the data types. For each data type, the following information is stored:

**identifier **Internal stable and perennial identifier.

**name **Expression commonly (and in general "officially") used to identify the data type.

**synonyms **Synonym(s) of the name (used for instance to store the expanded version of an acronym).

**definition **Short description of the data type, and the associated resources.

**identifier pattern **Regular expression of the identifiers used by this data type.

**official URL **URI used to identify the data type, following the Uniform Resource Locator syntax.

**official URN **URI used to identify the data type, following the Uniform Resource Name syntax.

**deprecated URIs **Deprecated versions of the URIs (which can be URLs or URNs).

**resources **Online data resources which provide datasets corresponding to the data type.

**- identifier **Internal stable and perennial identifier.

**- data entry **Physical address used to access a particular element stored by the data type.

**- data resource **Physical link to the main page of the resource.

**- information **Information about the resource.

**- institution **Name of the institution managing the resource.

**- country **Location of the institution managing the resource.

**- documentation **Link towards pieces of documentation about the data type.

**Figure 3:**
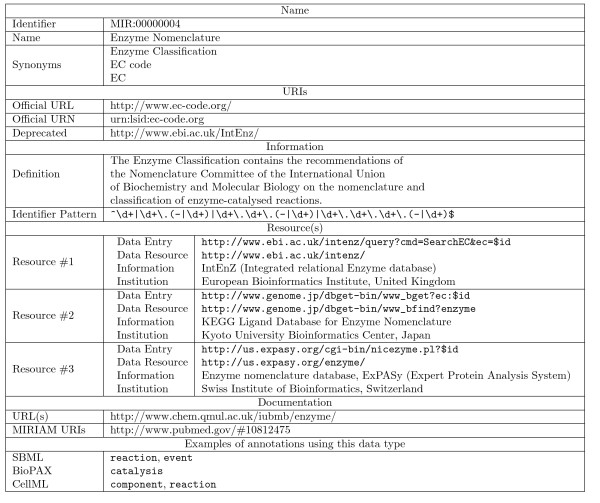
**Detail of an entry of MIRIAM Database**. The example represents the entry of Enzyme Nomenclature. Note the three alternative resources giving access to the same data types.

The first items represent general information. They are all mandatory, except the synonyms. The identifier is automatically generated during the submission process. It is perennial and stable. It varies from MIR:00000001 to MIR:00099999. An example of MIRIAM Database entry, describing the Enzyme Nomenclature is presented on Figure 3.

At least one official URI (whether it is a URL or a URN) needs to be provided for each data type. No more than one official URL and one official URN can be provided for a given data type. It may happen, although it should be rare, that data types merge, or an URI needs to be changed for various reasons. *MIRIAM URIs *are unique and persistent. Accordingly the root URI defining the data type must also be unique and persistent. It cannot be deleted, only deprecated. Deprecated URIs are stored to allow backward compatibility with models annotated using old identifiers, so that their annotation does not need to be rewritten. It is important to notice that the URI used to describe a data type is not a valid physical address. It is only an identifier and it should not be used to try to access a dataset on the Internet, whether using a Web browser or Web Services. If it happens to also be a valid physical address, this physical resource should be disregarded for MIRIAM purposes.

A resource is a service providing datasets corresponding to a data type. It could be a database accessible online through a Web-based interface, or a series of datasets available through FTP, etc. Several resources may exist for a given data type. Some are pure mirrors, but others may provide a different datasets, or datasets with slightly different metadata. A data type is always linked to at least one resource. Each resource is described by a stable and perennial identifier. It varies from MIR:00100001 to MIR:00199999. Documentation about a data type can be added as a full physical address (URL) or just as a *MIRIAM URI *(example: pubmed.gov/#16333295). The second choice is favoured to avoid any problem of resources unreachable in the future (it only relies on *MIRIAM Resources*).

### MIRIAM Web Services

*MIRIAM Resources *provide several resolution and conversion services, such as retrieving the information stored about a data type, generating a *MIRIAM URI *from a data type name and the identifier of a dataset, resolving all the physical locations corresponding to a *MIRIAM URI*, etc. *MIRIAM Resources *are not designed to be end-user software, but rather tools used by other programs via application-to-application communications. We provide a Web interface to perform queries on the database only as a demonstration of what *MIRIAM Web Services *can offer.

On the contrary, the programmatic access to *MIRIAM URI *is the "raison d'être" of *MIRIAM Resources*. MIRIAM requires to annotate quantitative models using standard URIs, that are perennial and shield the user from the resources distributing the datasets. A software developer, working for instance on a modelling environment or a simulation software, cannot develop support for all the possible Web Services offered by the data-providers in life-science. This developer would not even know which data types would be used by the end users to annotate their models, or would be present in the annotation of models imported. A resolving system had necessarily to be unique. Furthermore, there is not a single source of information for a given data type. For instance UniProt is accessible through the EBI (UK), the SIB (Switzerland) and the PIR (USA). Gene Ontology is available through dozens of resources around the world.

We offer a programmatic access through the Internet to *MIRIAM Database *via Web Services [31]. They are based on Simple Object Access Protocol (SOAP) [32], which is itself based on XML [33]. A public definition, which fully describes the methods provided, is available using the Web Services Description Language (WSDL) [34], also an XML-based language. The choice for an access based on SOAP, instead of other solutions, like Common Object Request Broker Architecture (CORBA) [35] or Distributed Component Object Model (DCOM) [36], relies on the fact that we wanted a standard, interoperable, reliable and easy to develop solution.

The interoperability is brought by all the protocols used: they are standards, mainly created by the World Wide Web Consortium (W3C). Moreover, all the messages are sent using the HTTP protocol [37], therefore, the access through firewalls is possible, without any special configuration. Finally, the success of SOAP-based Web Services [38] over the last half-decade means that software exist to make the development of MIRIAM clients very easy.

### MIRIAM Library

In order to encourage a rapid and widespread usage of *MIRIAM Web Services*, it was important to decrease the amount of work necessary to implement clients. The creation of a library, written in Java, was undertaken for that purpose. The package distributed comprises a precompiled library (jar) (running on all operating systems with a Java Virtual Machine available) and the source code. It is available from the MIRIAM project on SourceForge.net [39], the world's largest open-source software repository and project hosting service, as well as from the *MIRIAM Resources *pages at the EMBL-EBI Web site.

Two versions of the library are available: a standalone version, which does no needs any extra software to function properly, and another version, lighter, without all the dependencies (such as Apache Axis [40], Web Services Description Language for Java Toolkit (WSDL4J) [41] ...).

### MIRIAM Web Application

*MIRIAM Web Application *is the most visible part of *MIRIAM Resources*. It is a traditional Web application, based on the 1.4 Java 2 Platform Enterprise Edition (J2EE) technologies [42] (such as JavaServer Pages, Servlets ...).

No special framework (like Struts, Spring or Shale) was used in the development, but the internal structure of the application follows the Model-View-Controller (MVC) design pattern [43]. Moreover, a Servlet Controller has been created to handle all the requests. The application runs inside a Apache Tomcat Web container [44], version 5.0. Several other tools from the Apache Software Foundation are used, like Log4j [45] or Database Connection Pooling (DBCP) [46].

The application allows users to browse and query *MIRIAM Database*, submit new data types for inclusion in the database, export the whole content of the database and access all the information about the project (see the left-menu on Figure 1). The inclusion of new data types submitted through the interface depends on validation by members of the MIRIAM-team after verification that the submission fulfils MIRIAM requirements. In order to allow a dynamic display of the query interface, Asynchronous JavaScript and XML (AJAX) [47] has been used, via the library AjaxTags [48].

## Utility and Discussion

### MIRIAM Resources

A vast number of biological data resources and services have arisen over the last decades. However, whether they are located in bioinformatics "hubs" (NCBI, EBI ...) or distributed, their structure and mode of access is always specific. Past the institution front-page, there is little or not unification or standardisation of access to the data.

*MIRIAM Resources *enable computational systems biologists to access them using a unified scheme. Basically it is both an identifier scheme registry and a resolution service. It provides several services to the user, mainly dealing with generation (and storage) of URIs and retrieval of physical data from those URIs. One of the core features is to provide a unified interface to particular pieces of knowledge, regardless of the specifics of the sources. *MIRIAM Resources *can be considered as an interoperability framework for scientific collaboration on computational modelling [49].

### Curator's point of view

A model curator is a person who encodes, in a standard description format, a model created and described by somebody else, or corrects a model already encoded. For those curators (or even for the model creators, who are the people who initially designed the model), there is a need to put additional information on top of the model structure and mathematics. Whatever the format used to encode the model (SBML, CellML, BioPAX, MML, VCML ...), all the components of the model must be unambiguously identified.

Accordingly, *MIRIAM Standard *requires that each model constituent is linked to relevant entries in existing freely accessible resources ("External data resources annotation" in the main publication of MIRIAM). One way for a model to be declared MIRIAM compliant is to be accompanied by *MIRIAM URIs *linked to all the components.

The annotation of a model is a tedious but enlightening process. It is nevertheless much easier when coupled with the encoding or curation of the model. Indeed a curator had to already acquire a deep understanding of all the components of a model in order to correct its syntax and semantics. Therefore, the only thing needed is to use a model edition software to generate the appropriate URIs, such as SBMLeditor [50], based on the knowledge of a relevant accession for a given data type. Of course, this is possible only if the tool uses the method getURI() of *MIRIAM Web Services *(see below) or has a local version of *MIRIAM database*.

### Developer's point of view

The developer of a software to be used in computational systems biology will have to import models already encoded. If an interface to display them is to be created (Web-based or rich client), one needs to convert all the *MIRIAM URIs *for instance into physical addresses, which can be used to recover the knowledge stored in the entities pointed to by the annotations. The conversion from *MIRIAM URIs *to physical addresses can be done using the getDataEntries() method of *MIRIAM Web Services*.

### Current status and future developments

A fully functional version of *MIRIAM Resources *is already available online, providing all the services described in this article. Around forty different data types are currently recorded. Several projects already use *MIRIAM Resources *to resolve their annotation, such as BioModels Database [6] or the E-MeP project [51]. As the adoption of *MIRIAM Resources *spread in the community, the number of data types should grow accordingly.

With the increase of the usage of the resources, new needs will necessarily appear. New methods will be developed and added, via new releases, to the application. Users are encouraged to provide new data types as well as ideas to improve the resources. Another way of improving the data already stored is to provide, in addition to the addresses of Web pages presenting information about a relevant dataset, a programmatic access to the dataset itself (for instance via Web Services). Therefore, a wider range of applications would be able to retrieve the information.

*MIRIAM *and *MIRIAM Resources *were born in the field of Computational Systems Biology, in order to fulfil the needs of a better annotation of biochemical models. Nevertheless the current tools can be used in many other fields where similar issues exist: to identify datasets and be able to retrieve them consistently via a network. This is why the source code of the whole project (including the Web application, the Web Services and the library) is released under the terms of the GNU General Public License. Therefore, everybody is able to setup its own local resource to manage the data types they use and need.

## Conclusions

The project has now reached a fully functional and stable state. Therefore *MIRIAM Resources *can be safely adopted by model databases and software projects. As an example, it is currently used by BioModels Database to process the annotation of the models into relevant hyperlinks. It is also used by SBMLeditor for the creation of models compliant with *MIRIAM *in SBML. We hope that this work will help the adoption of MIRIAM as a standard rather than a mere set of guidelines, by providing tools to allow the community to easily create and annotate *MIRIAM *compliant models.

## Availability and requirements

*MIRIAM Resources *are accessible on the EMBL-EBI Web site, at the following address: .

The source code of the whole project (*MIRIAM Web Services*, *MIRIAM Library *and *MIRIAM Web Application*) is currently available under the GNU General Public License (GPL) and can be downloaded at: .

## Authors' contributions

NLN listed feature requirements and gave advice on the overall direction of the project. CL developed all the components of *MIRIAM Resources*. Both contributed, with the help of the community, to the creation of the current content of the database.
